# Community acceptance of Ivermectin mass drug administration for malaria in Southern Thailand

**DOI:** 10.1038/s41598-025-14575-y

**Published:** 2025-08-08

**Authors:** Pyae Linn Aung, Piyarat Sripoorote, Nattawan Rachaphaew, Jetsumon Sattabongkot, Daniel M. Parker, Wang Nguitragool, Suparat Phuanukoonnon

**Affiliations:** 1 Mahidol Vivax Research Unit, Faculty of Tropical Medicine, Mahidol University, Bangkok, Thailand; 2 Department of Population Health and Disease Prevention, University of California, Irvine, USA; 3 Department of Epidemiology and Biostatistics, University of California, Irvine, USA; 4 Department of Molecular Tropical Medicine, Faculty of Tropical Medicine, Mahidol University, Bangkok, Thailand; 5 Department of Social and Environmental Medicine, Faculty of Tropical Medicine, Mahidol University, Bangkok, Thailand

**Keywords:** Acceptance, Ivermectin, KAP, Malaria, MDA, Thailand, Vector control, Health care, Risk factors, Malaria

## Abstract

Thailand’s goal to eliminate malaria by 2030 faces significant challenges, in part due to inadequate vector control measures. Innovative strategies, such as mass drug administration (MDA) of ivermectin, have shown promise in improving vector control efforts. This study aims to assess the factors influencing the acceptance of ivermectin MDA among residents in southern Thailand. In 2022, a cross-sectional study was conducted in three districts of Surat Thani Province, southern Thailand, where an ivermectin MDA program was planned. Quantitative data were collected through structured surveys administered to randomly selected household heads using a standardized questionnaire. Descriptive statistics and multiple logistic regression models were employed to identify factors associated with the reported acceptance of ivermectin MDA. A total of 391 participants were surveyed, with the majority (96.4%) expressing acceptance of the planned ivermectin MDA. Forest-related workers (aOR: 4.2, 95% CI: 1.1–16.1) and those who believed malaria could be eliminated from their villages (aOR: 9.1, 95% CI: 2.8–29.9) were more likely to exhibit higher levels of acceptance. However, according to programmatic records, only 59.0% of the 3,137 eligible individuals completed all three rounds of ivermectin MDA. Key barriers to participation included absence from the village for unspecified reasons and reluctance to take the drug, particularly in later rounds. There was a noticeable gap between reported acceptance and actual participation in the ivermectin MDA. Targeted efforts to engage forest-related workers are crucial to maintain high participation rates. Strengthening community engagement by emphasizing the risks and benefits of ivermectin, outlining safety measures, and raising awareness about malaria prevention and control are crucial for improving MDA uptake.

## Background

The Greater Mekong Subregion (GMS) has been grappling with malaria for decades, with drug-resistant malaria parasites complicating control efforts^1,2^. Among the six countries in the region, Thailand has made significant progress toward controlling malaria and is committed to achieving zero local malaria transmission by 2030. Despite these efforts, approximately 17,000 malaria cases were reported in 2023, representing a sevenfold increase from 2021, with nearly 42% classified as imported^3,4^. Border malaria remains a significant challenge for Thailand, particularly in regions like Tak Province, near the Thailand-Myanmar border^5–7^. Therefore, maintaining a robust surveillance system capable of detecting malaria cases and ensuring timely vector control measures are essential to interrupt transmission.

Malaria transmission is influenced by the complex interplay between the host, vector, and parasite. The *Anopheles* mosquitoes that vector malaria in Southeast Asia thrives in forested areas with abundant vegetation, making vector control particularly challenging^8^. Due to the difficulties in controlling larvae, vector control in Thailand primarily relies on insecticide-treated nets (ITNs) and indoor residual spraying (IRS)^9^. However, a study in northwestern Thailand found that only 53.1% of participants used ITNs correctly on a daily basis, highlighting the need for targeted interventions to improve ITN use, particularly among high-risk groups^10^. Furthermore, insecticide resistance poses a major ecological challenge, undermining the success of malaria control efforts^11^. Studies have shown that major vectors, such as *Anopheles minimus*, *An. dirus*, and *An. maculatus* have developed resistance to several insecticide classes, including pyrethroids, organophosphates, and carbamates^12–14^. Continuous monitoring and management of insecticide resistance in malaria vector populations are crucial, alongside innovative approaches to expedite effective vector control measures.

Ivermectin, a medication traditionally used to treat parasitic infections in humans and animals, has shown promise in malaria control and warrants further investigation as a potential tool for reducing malaria transmission^15,16^. One study found that ivermectin has a mosquito population reducing effect, killing *Anopheline* vectors that feed on treated individuals. Mass drug administration (MDA) of ivermectin has also demonstrated potential in reducing malaria incidence^17^. A modeling study showed that three rounds of ivermectin MDA could reduce clinical malaria incidence by up to 71% in both high-transmission and resurgence-preventing areas^18^. Another study recommended ivermectin as a strategy to reduce malaria transmission, particularly among individuals who rarely use ITNs, by increasing malaria vector mortality^19^. However, further research is needed to determine the optimal dosage, timing, and delivery mechanisms. Following recommendations from an entomological study in Southern Thailand which reported a high prevalence of primary malaria vectors in Surat Thani Province, a mass ivermectin treatment campaign was proposed^20^.

Effective MDA programs depend on high levels of community participation^19^. Even minimal non-participation can undermine potential benefits, such as the permanent elimination of malaria or temporary reductions in infection prevalence. Therefore, understanding factors that influence participation is crucial to the success of MDA programs^21^. Research indicates that knowledge, attitudes and practice (KAP) regarding malaria prevention and control can increase the willingness to participate, while misconceptions about malaria transmission and concerns over medication side effects may deter participation^22–24^. Thus, implementing MDA must be carefully planned and communicated to communities to ensure trust and high participation^19^. Barriers and enabling factors may affect participation at different stages of ivermectin MDA programs. However, there is a lack of comprehensive studies explicitly examining the factors influencing participation in ivermectin MDA for malaria control in Thailand or elsewhere. Therefore, this study aims to investigate people’s preexisting KAP concerning malaria and assess their reported acceptance of the forthcoming ivermectin MDA campaign in southern Thailand.

## Methods

### Study design and location

**Fig. 1:**
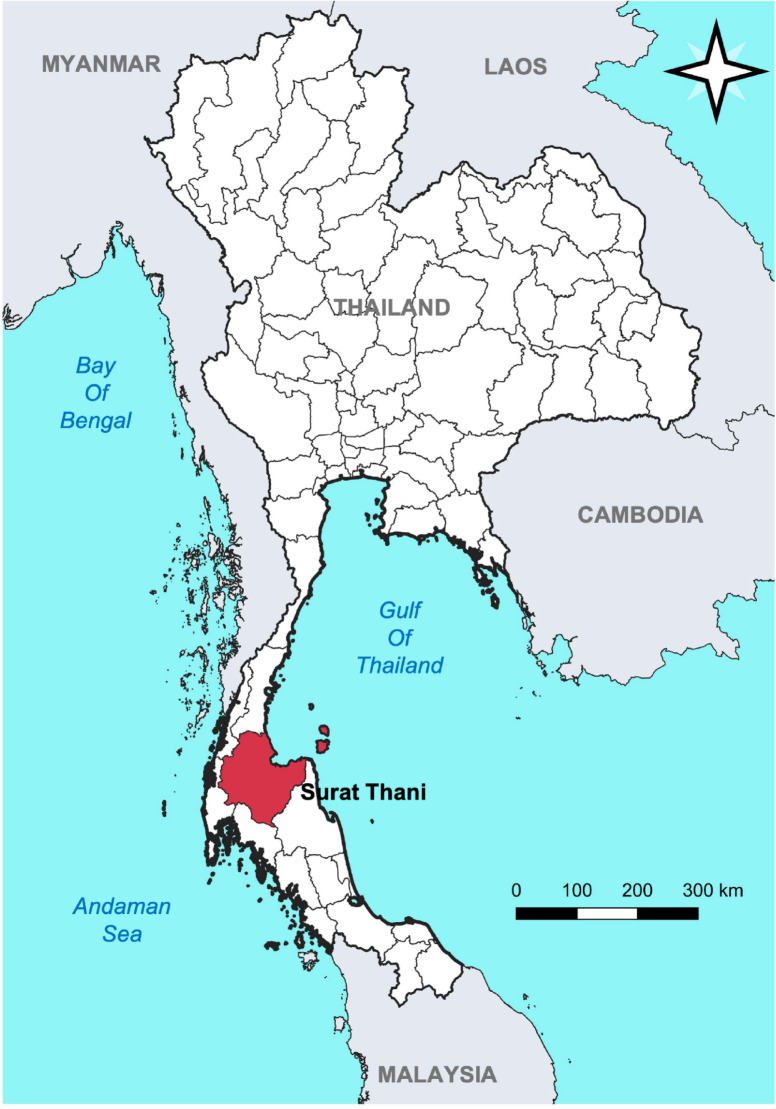
Study location map. The map was generated using QGIS version 3.34.2 (Prizren).

This community-based cross-sectional study collected quantitative data in late 2022. Among Thailand’s 76 provinces, Surat Thani, located in the southern region, was deliberately selected for its suitable characteristics, including environmental factors, malaria incidence, human occupations, and vector distribution. The area is predominantly covered by rubber and palm plantations. The primary malaria vector in this region is *An. minimus*, which is highly susceptible to ivermectin^20,25,26^. Additionally, *P. falciparum*, a parasite species without relapse, is the major malaria strain in the area, making it an ideal candidate for testing the effects of ivermectin MDA. While malaria cases have declined in Surat Thani, the area remains receptive to transmission due to ongoing occupational risks and the presence of competent malaria vectors^4^. In Surat Thani province, nine villages from three districts were purposely selected as study areas based on baseline population density, occupational distribution, and overall feasibility, in line with recommendations from provincial malaria stakeholders and human migration patterns (Fig. 1).

### Sample size estimation and sampling

The required sample size across the study site was calculated using the formula for single cross-sectional cluster survey^27^ with a reference proportion from a previous study in India, where approximately 68.3% of participants refused to accept ivermectin MDA^28^. Allowing for a 5% margin of error and an estimated design effect of 2.5, the calculated sample size was 332. After factoring in a 15% non-response rate, the final required sample size was at least 382.

Before initiating the ivermectin MDA trial, a baseline population census was conducted in all nine selected villages. From the final population lists, household heads were listed separately, and systematic random sampling was applied. In cases where the selected individual was unavailable, an alternative household member was selected, or another randomization was conducted if the entire household was absent. An average of 40 to 45 participants from each village were recruited, representing approximately 15—20% of all households in the planed MDA villages. Household heads, male or female, aged 18 years or older and residing in the village for more than one year were eligible to participate. Individuals unable to communicate due to illness or those unwilling to participate were excluded. These criteria ensured that respondents were familiar with the local context and health services. Ultimately, 391 participants were recruited and included in the final analysis.

### Data collection

Data were gathered using a standardized and validated questionnaire, developed in English, and based on previous studies^22,23^ and relevant literature^29^. The questionnaire was then translated into Thai using a back-translation method by two independent social science researchers.

The questionnaire comprised five parts. The first part assessed each participant’s general characteristics, such as age, gender, education, occupation, marital status, family size, their role in the family, residential status, and history of malaria infection. The second part explored participants’ knowledge about malaria, including its transmission, symptoms, diagnosis, treatment, and reinfection. This section contained nine major questions, each with multiple predefined choices, with a total possible score of 36 points. The third part assessed attitudes toward malaria, consisting of 15 mini-statements covering the perceived severity, causation, compliance with treatment, and prevention. Each participant could score a maximum of 45 points in this section. The following fourth section evaluated participants’ practices related to malaria prevention and treatment, including their experience with taking prescribed antimalarial drugs, use of prevention methods, and beliefs about the possibility of eliminating malaria from the village. Each participant could receive a maximum score of 20 points.

Before the final section of the survey, each participant was provided with a succinct overview of the upcoming ivermectin MDA campaign, detailing the recommended drug dosages, duration of treatment, frequency of blood sample collection, and the potential benefits of participation for malaria control. The last part of the survey explored the relative acceptability of participants towards forthcoming ivermectin MDA, with options to answer “Accept”, “Not sure”, or “Not accept”.

To compare reported acceptance with actual participation in the ivermectin MDA, each surveyed individual was checked against the list of MDA participants at the end of each round. There were three consecutive rounds of MDA, and in each round, eligible individuals who did not participate were asked the main reasons for non-participation, either directly from their family members or neighbors, as appropriate. While not part of the primary survey, this information was included to assess the discrepancy between intention and behavior at the community level. This component was descriptive and did not involve individual linkage with the survey responses. The inclusion of these program records reflects a complementary data source rather than a mixed-methods or secondary data analysis.

To conduct data collection, 3 to 4 healthcare staff and 4 to 5 village malaria volunteers were recruited from each selected study area. A one-day training session was held, covering questionnaire administration, response recording, and ethical considerations. Trained data collectors administered structured surveys to participants in the selected villages. Each survey session typically lasted no more than 20 min. The research team supervised data collection, checked for any missing data or discrepancies daily and made necessary corrections.

## Data entry and analysis

The data were entered into Microsoft Excel, coded, and transferred to the Statistical Package for the Social Sciences (IBM SPSS Statistics for Macintosh, version 28, IBM Corp., Armonk, NY, USA) for further analysis.

Descriptive statistics (frequencies and percentages) were used to present participant characteristics. For the knowledge and practice assessments, participants received one point for each correct answer and zero for incorrect or no responses. Attitude responses were measured on a 3-point Likert scale. Participants received 3 points for “Agree,” 2 points for “Not sure,” and 1 point for “Disagree” on positive statements. The scoring was reversed for negative statements. Total scores for each participant were combined separately for KAP and categorized as “good” or “poor” by applying a criterion of < mean or ≥ mean. The overall KAP levels were visualized using bar graphs. The reported acceptability of ivermectin was presented using a pie chart.

To compare reported acceptability with actual ivermectin MDA participation, the number of participants completing each round was analyzed descriptively. For eligible individuals who did not fully participate, primary reasons for non-completion were recorded.

Multivariable logistic regression models were used to identify factors associated with poor KAP levels, and ivermectin MDA acceptability. Adjusted odds ratios (aOR) and 95% confidence intervals (CIs) were calculated, with all independent variables included in the regression models, regardless of their initial associations, to develop a comprehensive model. Associated factors were identified based on the 95% CIs, with statistical significance inferred when the interval did not include the null value of 1.

## Results

### General characteristics of participants

The general characteristics of 391 study participants were assessed. The findings revealed that the majority of the participants were female (50.6%) and had completed primary school education (60.1%). More than one-third (44.5%) were household heads. Over half of the participants (51.9%) had migrated to the area over a decade ago, and a significant portion (59.6%) had previously experienced malaria. Most participants were aged 36 years or older (79.3%). Furthermore, nearly 90% were engaged in farming or gardening (92.8%) and the majority were married (90.3%). Most participants lived in households with 1 to 6 family members (89.0%), with an average household size of 4 and the median also being 4 (Table 1).

**Table 1:** General characteristics of the respondents (*n* = 391).

Characteristic	Frequency (*n*)	Percentage (%)
**Gender**		
Male	193	49.4
Female	198	50.6
**Age (years)**		
18 to 35	81	20.7
36 to 50	154	39.4
> 50	156	39.9
Mean ± SD (Min, Max)	46.5 ± 12.2 (19, 80)	
**Education**		
Elementary school	235	60.1
High school	117	29.9
Diploma	21	5.4
Bachelor’s or higher	17	4.3
Other (vocational training)	1	0.3
**Occupation**		
Housewife	7	1.8
General worker	12	3.1
Own business	5	1.3
Civil servant	2	0.5
Agriculture	2	0.5
Farmers/gardeners	363	92.8
**Marital status**		
Single	27	6.9
Married	353	90.3
Widowed/divorced/separated	11	2.8
**Total family members**		
1 to 3	174	44.5
4 to 6	174	44.5
> 6	43	11.0
**Role of the respondent in the family**		
Household head	174	44.5
Husband/wife	153	39.1
Other members	64	16.4
**Residential status**		
Since birth	141	36.1
Moved from another location (within 10 years)	47	12.0
Moved from another location (over 10 years)	203	51.9
**Previous attacks of malaria**		
Never	134	34.3
Ever	233	59.6
Don’t know	24	6.1

### Knowledge about malaria

Participant’s knowledge regarding malaria was thoroughly evaluated. Nearly all participants (98.5%) identified mosquito bites as the primary cause of malaria. However, misconceptions persisted, with some believing that drinking stagnant water (29.9%) or living in the forest (37.3%) could cause malaria. Furthermore, a significant majority (92.3%) correctly identified the mosquito species responsible for malaria transmission. In term of diagnosis, approximately half of the participants consulted healthcare professionals (46.5%) or underwent blood tests (57.8%) to confirm malaria infection. However, about one-third (38.9%) relied on recognizing symptoms to determine if they had malaria. Most participants correctly identified fever (85.2%), chills and rigor (96.4%), and headache (82.6%) as the main symptoms of malaria. Nearly all participants (98.7%) adhered to prescribed treatment from healthcare providers. While most participants (96.4%) advised using mosquito nets as a preventive measure, a small fraction believed that avoiding drinking water in the forest (4.9%) could prevent malaria. Additionally, the majority (85.9%) suggested using mosquito repellents to prevent mosquito bites. Participants were well aware of the possibility of malaria reinfection (96.2%) even after previously being cured. A significant proportion (41.9%) acknowledged that persistent malaria-like symptoms, even after treatment, might be due to poor treatment adherence (58.1%) or severe infection (46.0%) (Table 2).

**Table 2:** Knowledge about malaria (*n* = 391).

Description	Yes	
	*n*	%
**How is malaria transmitted?**		
Hard-working	1	0.3
Living in the forest	146	37.3
Drinking stagnant water	117	29.9
Staying with sick people	59	15.1
Biting of mosquito	385	98.5
Using water in the forest	19	4.9
**Which mosquito carries the malaria parasites?**		
*Aedes*	25	6.4
*Anopheles*	361	92.3
*Culex*	3	0.8
**How could you know you had malaria?**		
By consultation with healthcare officers	182	46.5
Having a blood test	226	57.8
Ask other people with malaria experience	3	0.8
According to the symptoms of illness	152	38.9
**What are the symptoms of malaria?**		
Fever	333	85.2
Chills and rigor	377	96.4
Fatigue	63	16.1
Loss of appetite and weight loss	51	13.0
Abdominal pain	151	38.6
Nausea and vomiting	109	27.9
Headache	323	82.6
**How should malaria be treated?**		
Taking herbal medicines	29	7.4
Getting a good rest	39	10.0
Having prescribed medications from healthcare officers	386	98.7
**How should malaria be prevented?**		
Avoid drinking water in the forest	19	4.9
Sleeping under a mosquito net	377	96.4
Preventing mosquito bites	214	54.7
Boosting the immune system	69	17.6
Receiving regular medical check-ups	5	1.3
Taking malaria chemoprophylaxis	27	6.9
Using mosquito repellents	336	85.9
**Once a person is relieved from malaria**,** can they be reinfected again?** (Yes)	376	96.2
**Can anyone still suffer malaria even after a course of treatment?** (Yes)	164	41.9
**What are the factors associated with incurable malaria?**		
Poor compliance of patients with malaria	227	58.1
Malaria itself is incurable	7	1.8
Severe infection	180	46.0
Antimalarial drug resistance	65	16.6

### Attitudes toward malaria

Table 3 presents the participants’ attitudes toward malaria. Nearly all acknowledged the severity of malaria and agreed that they were susceptible to infection (98.2%). They also agreed that malaria could be fatal (91.6%) and that wealth status was unrelated to the risk of infection (94.9%). Most participants recognized the importance of completing treatment (93.9%) and emphasized the safety of antimalarial drugs (43.5%). More than two-thirds of participants supported preventive measures such as IRS (84.7%), while some (35.8%) were aware that malaria was not confined to specific areas. However, a significant number of participants (46.3%) were uncertain about the potential adverse effects of combining malaria medication with foods such as durian.

**Table 3:** Attitudes towards malaria (*n* = 391).

Statement	Agree *n* (%)	Not sure *n* (%)	Disagree *n* (%)
Malaria can cause death.	358 (91.6)	16 (4.1)	17 (4.3)
No local people died from malaria. ^a^	166 (42.5)	135 (34.5)	90 (23.0)
Everyone is susceptible to malaria.	384 (98.2)	5 (1.3)	2 (0.5)
Rich people do not get malaria. ^a^	16 (4.1)	371 (94.9)	4 (1.0)
Burmese workers are more prone to malaria than Thai people.	202 (51.7)	25 (6.4)	164 (41.9)
Children with malaria are more severe than adults.	223 (57.0)	15 (3.8)	153 (39.1)
Drinking alcohol kills malaria parasites. ^a^	35 (9.0)	354 (90.5)	2 (0.5)
Free malaria treatment allows patients not to take medicine. ^a^	82 (21.0)	280 (71.6)	29 (7.4)
The patient must take the total medications for a radical malaria cure.	367 (93.9)	7 (1.8)	17 (4.3)
Taking malaria medicines may cause side effects. ^a^	190 (48.6)	31 (7.9)	170 (43.5)
It is forbidden to treat malaria together with fruits, such as durian. ^a^	181 (46.3)	119 (30.4)	91 (23.3)
Malaria prevention is the task of healthcare officers only. ^a^	34 (8.7)	180 (46.0)	177 (45.3)
Indoor residual spraying can prevent malaria.	331 (84.7)	14 (3.6)	46 (11.8)
Malaria is a matter of fate. ^a^	1 (0.3)	390 (99.7)	0 (0)
Malaria is a localized problem. ^a^	116 (29.7)	135 (34.5)	140 (35.8)

^a^ Negative statement.

### Malaria prevention and treatment practices

Table 4 outlines the study participants’ practices regarding malaria prevention and treatment. More than three-quarters of participants (76.0%) had experienced malaria before the study. Among those with a history of malaria, the majority sought treatment at hospitals (72.1%) or malaria clinics (57.6%) and completed their prescribed treatment (99.3%). However, a proportion (17.9%) admitted to stopping their medication once symptoms subsided. Regarding preventive measures, participants mainly used bed nets (94.1%) and mosquito repellents (88.8%). Other strategies, such as burning leaves and wood, to produce smoke to repel mosquitoes (55.2%) or using mosquito coils (18.9%), were also employed. Personal protective measures, such as wearing long sleeves (36.5%) or insecticide-treated clothing (23.5%), were less commonly practiced. Most participants believed that malaria could be eradicated from their villages (76.7%) through collaboration between healthcare workers (80.1%) and community members (87.5%).

**Table 4:** Practice on malaria prevention and treatment (*n* = 391).

Description	Yes	
	*n*	%
**Have you or your family members ever suffered from malaria?**	297	76.0
**Where did you go for the treatment?** (*n* = 297)		
Malaria clinic	171	57.6
Hospital	214	72.1
Private clinic	1	0.3
**How did you take the prescribed medicines? (***n* = 297)		
Stopped taking the medicines soon after the symptoms were relieved	2	0.7
Took complete course of treatment given by the health officers	295	99.3
**What do you think is why people are not taking the complete course?**		
Symptoms have disappeared	70	17.9
Suffered side effects from the medicines	24	6.1
Keeping the drugs for future use	9	2.3
**Are you practicing something to prevent mosquito bites?**	375	95.9
**How do you prevent it?** (*n* = 375)		
Sleeping under bed nets	353	94.1
Wearing long sleeves clothing	137	36.5
Using mosquito repellents	333	88.8
Burning mosquito coils	71	18.9
Burning leaves and wood to produce smoke to repel mosquitoes	207	55.2
Wearing insecticide-treated clothes	88	23.5
Other (using air-cooling fan)	17	4.5
**Do you believe malaria can be eradicated from your village?**	300	76.7
**Who should be involved in eliminating malaria from the village?**		
Healthcare officers	313	80.1
Villagers	342	87.5
Others (community leaders)	9	2.3

### Overall knowledge, attitude, and practice levels and associated factors with poor levels

**Fig. 2:**
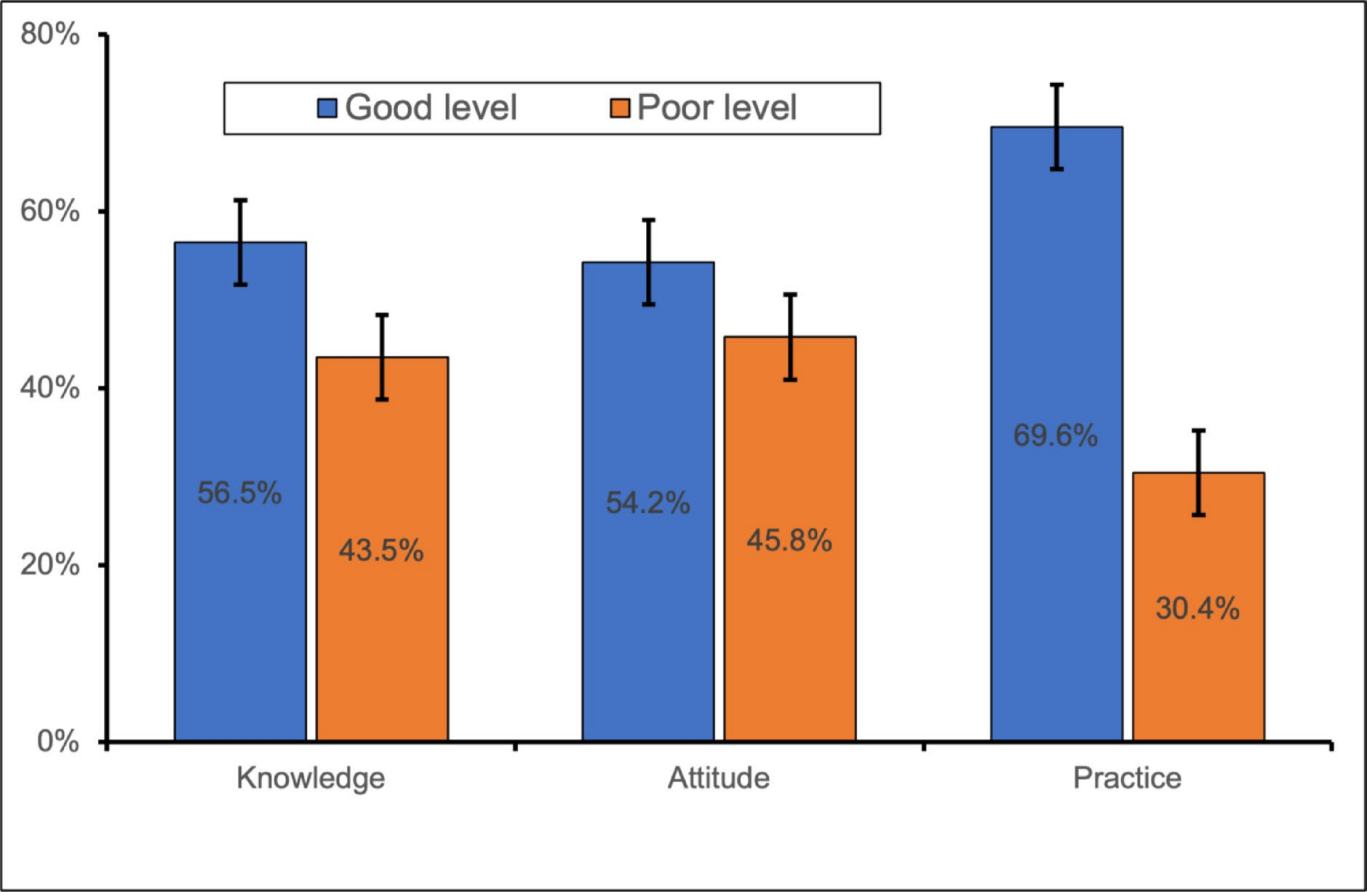
Overall malaria knowledge, attitude, and practice levels (*n* = 391).

Participants’ KAP regarding malaria prevention and treatment were categorized as “good” or “poor” based on their scores. The results revealed that over half of the participants demonstrated a good level of knowledge (56.5%) and attitudes toward malaria prevention and treatment (54.2%). Additionally, a significant majority of the participants (69.6%) had good preventive and treatment practices (Fig. 2).

Table 5 presents an analysis of the associations between the general characteristics of study participants and their KAP levels regarding malaria prevention and treatment. The findings revealed that gender, marital status, educational attainment, and role in the family did not significantly affect knowledge levels. However, participants aged 36 to 50 years (53.9%), engaged in non-forest-related occupations (57.7%), with fewer than six family members (46.0%), who had recently moved (46.8%), or who had uncertain malaria histories (70.8%) were more likely to exhibit poor knowledge levels compared to other groups. Furthermore, the logistic regression indicated that participants aged 36 to 50 years (aOR: 1.7, 95%CI: 1.0–2.9) and those without a history of malaria (aOR: 2.2, 95%CI: 1.4–3.4) or with uncertain malaria histories (aOR: 4.6, 95%CI: 1.8–11.5) were more likely to have poor malaria knowledge.

**Table 5:** Associated factors with poor knowledge, attitudes and practices regarding malaria prevention and treatment (*n* = 391).

Factor	Poor knowledge		Poor attitudes		Poor practice	
	*n* (%)	aOR (95% CI)	*n* (%)	aOR (95% CI)	*n* (%)	aOR (95% CI)
**Gender**						
Male	83 (43.0)	1.0 (0.7, 1.4)	91 (47.2)	1.1 (0.8, 1.7)	55 (28.5)	0.8 (0.5, 1.3)
Female	87 (43.9)	Ref.	88 (44.4)	Ref.	64 (32.3)	Ref.
**Age (years)**						
18 to 35	33 (40.7)	Ref.	36 (44.4)	Ref.	24 (29.6)	Ref.
36 to 50	83 (53.9)	1.7 (1.0, 2.9)	71 (46.1)	1.1 (0.6, 1.8)	66 (42.9)	1.8 (1.0, 3.2)
> 50	54 (34.6)	0.8 (0.4, 1.3)	72 (46.2)	1.1 (0.6, 1.8)	29 (18.6)	0.5 (0.3, 1.0)
**Education**						
Elementary school	104 (44.3)	0.8 (0.4, 1.7)	106 (45.1)	0.5 (0.3, 1.0)	67 (28.5)	0.6 (0.3, 1.2)
High school	47 (40.2)	0.7 (0.3, 1.5)	49 (41.9)	0.5 (0.2, 1.0)	36 (30.8)	0.6 (0.3, 1.4)
Diploma or higher	19 (48.7)	Ref.	24 (61.5)	Ref.	16 (41.0)	Ref.
**Occupation**						
Non-forest-related	15 (57.7)	1.9 (0.8, 4.1)	20 (76.9)	4.3 (1.7, 11.0)	11 (42.3)	1.8 (0.8, 3.9)
Forest-related (Farmers/gardeners)	155 (42.5)	Ref.	159 (43.6)	Ref.	108 (29.6)	Ref.
**Marital status**						
Single	13 (48.1)	Ref.	11 (40.7)	Ref.	9 (33.3)	Ref.
Married	153 (43.3)	0.8 (0.4, 1.8)	161 (45.6)	1.2 (0.6, 2.7)	107 (30.3)	0.9 (0.4, 2.0)
Widowed/divorced/separated	4 (36.4)	0.6 (0.2, 2.6)	7 (63.6)	2.6 (0.6, 10.8)	3 (27.3)	0.8 (0.2, 3.5)
**Total family members**						
1 to 3	76 (43.7)	Ref.	78 (44.8)	Ref.	49 (28.2)	Ref.
4 to 6	80 (46.0)	1.1 (0.7, 1.7)	85 (48.9)	1.2 (0.8, 1.8)	51 (29.3)	1.1 (0.7, 1.7)
> 6	14 (32.6)	0.6 (0.3, 1.3)	16 (37.2)	0.7 (0.4, 1.5)	19 (44.2)	2.0 (1.0, 4.0)
**Role of the respondent in the family**						
Household head	82 (47.1)	1.4 (0.9, 2.2)	82 (47.1)	1.1 (0.7, 1.7)	42 (24.1)	0.6 (0.4, 1.0)
Husband/wife	59 (38.6)	Ref.	69 (45.1)	Ref.	51 (33.3)	Ref.
Other members	29 (45.3)	1.3 (0.7, 2.4)	28 (43.8)	1.0 (0.5, 1.7)	26 (40.6)	1.4 (0.8, 2.5)
**Residential status**						
Since birth	54 (38.3)	Ref.	59 (41.8)	Ref.	42 (29.8)	Ref.
Moved from another location (within 10 years)	21 (44.7)	1.3 (0.7, 2.5)	20 (42.6)	1.0 (0.5, 2.0)	16 (34.0)	1.2 (0.6, 2.5)
Moved from another location (over 10 years)	95 (46.8)	1.4 (0.9, 2.2)	100 (49.3)	1.4 (0.9, 2.1)	61 (30.0)	1.0 (0.6, 1.6)
**Previous attacks of malaria**						
Never	72 (54.1)	2.2 (1.4, 3.4)	54 (40.6)	0.8 (0.5, 1.2)	69 (51.9)	7.1 (4.3, 11.7)
Ever	81 (34.6)	Ref.	108 (46.2)	Ref.	31 (13.2)	Ref.
Don’t know	17 (70.8)	4.6 (1.8, 11.5)	17 (70.8)	2.8 (1.1, 7.1)	19 (79.2)	24.9 (8.7, 71.5)

aOR: adjusted odds ratio.

Regarding attitude levels, participants with higher education (61.5%), non-forested-related occupations (76.9%), and uncertain malaria histories (70.8%) exhibited poorer attitudes towards malaria prevention and treatment compared to other groups. Participants engaged in non-forest-related occupations (aOR: 4.3, 95%CI: 1.7–11.0) and those with uncertain malaria histories (aOR: 1.1, 95%CI: 1.1–7.1) were more likely to have poor attitudes.

For practice levels, participants aged 36 to 50 years (42.9%), with higher education (41.0%), non-forest-related occupations (42.3%), large families (44.2%), or no history of malaria (51.9%) or undetermined malaria histories (79.2%) exhibited poor practices. Three variables showed higher odds of poor practices: aged 36 to 50 years (aOR: 1.8, 95%CI: 1.0–3.2), large families (aOR; 2.0, 95%CI: 1.0–4.0), or no history of malaria (aOR: 7.1, 95%CI: 4.3–11.7), or uncertain malaria histories (aOR: 24.9, 95%CI: 8.7–71.5) (Table 5).

### Community acceptability of Ivermectin MDA and its associated factors

**Fig. 3:**
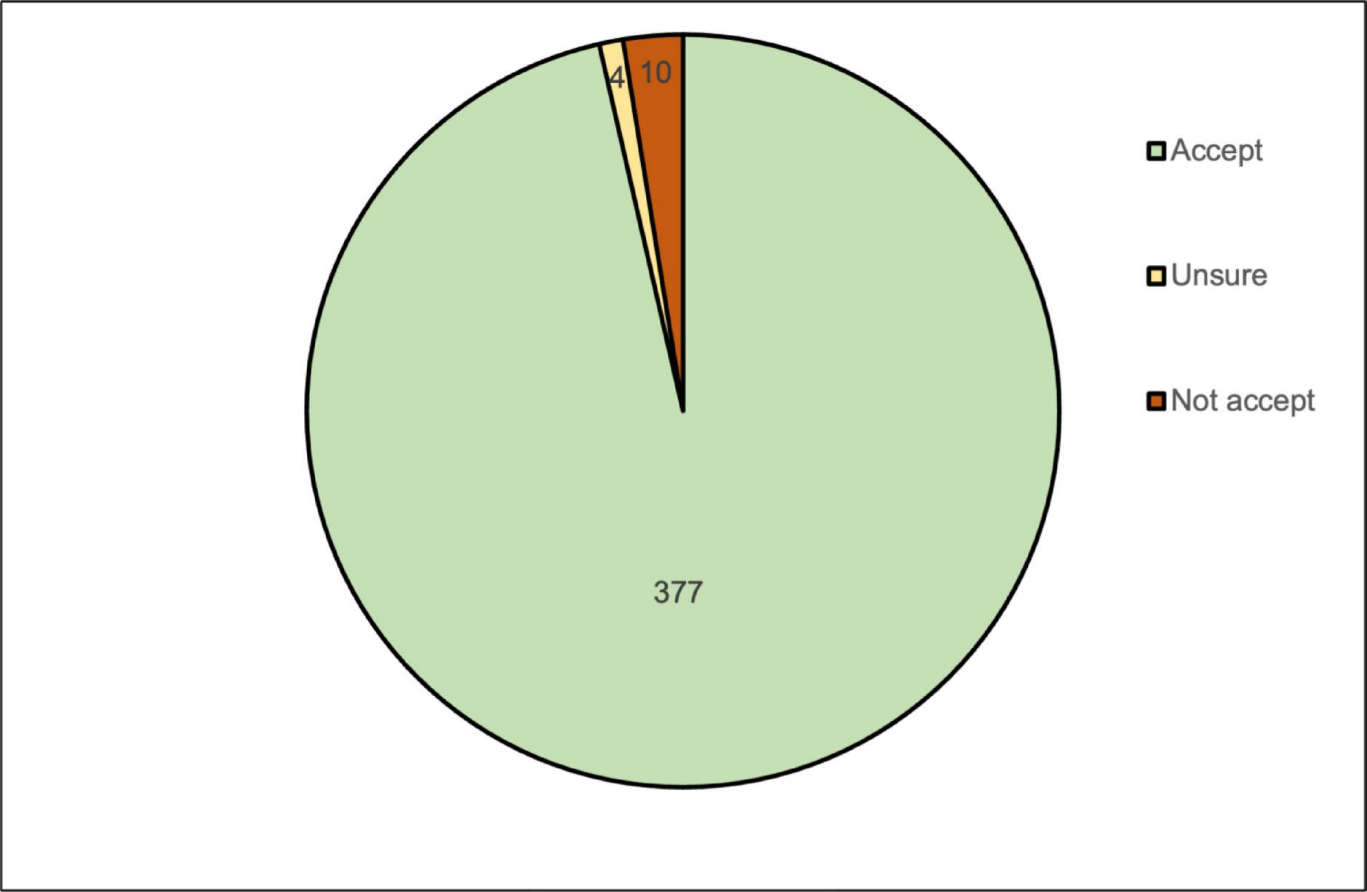
Reported community acceptability towards Ivermectin mass drug administration (*n* = 391).

This study assessed the acceptability of ivermectin MDA among study participants. The results showed that the vast majority (96.4%) were willing to participate in ivermectin MDA, while only a small proportion (2.5%) expressed reluctance (Fig. 3). Multivariable logistic regression analyses identified two variables significantly associated with higher acceptability: employment in forest-related occupations (aOR: 4.2, 95% CI: 1.1–16.1), and the belief that malaria could be eliminated from the village (aOR: 9.1, 95% CI: 2.8–29.9) (Table 6).

**Table 6:** Factors associated with reported acceptability to Ivermectin mass drug administration (*n* = 391).

Factor	Accept *n* (%)	Not accept *n* (%)	aOR (95% CI)
**Gender**			
Male	188 (97.4)	5 (2.6)	Ref.
Female	189 (95.5)	9 (4.5)	1.0 (0.3, 3.5)
**Age (years)**			
18 to 35	78 (96.3)	3 (3.7)	Ref.
36 to 50	151 (98.1)	3 (1.9)	1.9 (0.4, 9.8)
> 50	148 (94.9)	8 (5.1)	0.7 (0.2, 2.8)
**Education**			
Elementary school	225 (95.7)	10 (4.3)	Ref.
High school	115 (98.3)	2 (1.7)	2.6 (0.6, 11.9)
Diploma or higher	37 (94.9)	2 (5.1)	0.8 (0.2, 3.9)
**Occupation**			
Non-forest-related	23 (88.5)	3 (11.5)	Ref.
Forest-related (Farmers/gardeners)	354 (97.0)	11 (3.0)	4.2 (1.1, 16.1)
**Marital status**			
Single	25 (92.6)	2 (7.4)	Ref.
Married	342 (96.9)	11 (3.1)	2.5 (0.5, 11.8)
Widowed/divorced/separated	10 (90.9)	1 (9.1)	0.8 (0.1, 9.8)
**Total family members**			
1 to 3	168 (96.6)	6 (3.4)	Ref.
4 to 6	168 (96.6)	6 (3.4)	1.0 (0.3, 3.2)
> 6	41 (95.3)	2 (4.7)	0.7 (0.1, 3.8)
**Role of the respondent in the family**			
Household head	169 (97.1)	5 (2.9)	Ref.
Husband/wife	147 (96.0)	6 (3.9)	0.7 (0.2, 2.4)
Other member	61 (95.3)	3 (4.7)	0.6 (0.1, 2.6)
**Residential status**			
Since birth	137 (97.2)	4 (2.8)	0.7 (0.1, 6.8)
Moved from another place (within 10 years)	46 (97.9)	1 (2.1)	Ref.
Moved from another place (over 10 years)	194 (95.6)	9 (4.4)	0.5 (0.1, 3.8)
**Previous attacks of malaria**			
Never	127 (95.5)	6 (4.5)	Ref.
Ever	228 (97.4)	6 (2.6)	1.8 (0.6, 5.7)
Don’t know	22 (91.7)	2 (8.3)	0.5 (0.1, 2.7)
**Malaria knowledge**			
Good	214 (96.8)	7 (3.2)	1.3 (0.5, 3.8)
Poor	163 (95.9)	7 (4.1)	Ref.
**Attitudes toward malaria**			
Good	204 (96.2)	8 (3.8)	0.9 (0.3, 2.6)
Poor	173 (96.6)	6 (3.4)	Ref.
**Malaria prevention and treatment practices**			
Good	265 (97.4)	7 (2.6)	2.4 (0.8, 6.9)
Poor	112 (94.1)	7 (5.9)	Ref.
**Belief in malaria eradication from village**			
Yes	296 (98.7)	4 (1.3)	9.1 (2.8, 29.9)
No	81 (89.0)	10 (11.0)	Ref.

aOR: adjusted odds ratio.

### Reasons for non-participation in each round of MDA

In addition to the primary survey, the study team reviewed official MDA attendance records for all 3,137 eligible individuals in the nine study villages. These programmatic data provided insight into the actual coverage achieved during each round of ivermectin MDA. Participation declined across the rounds: 78.6% in round 1, 69.7% in round 2, and 70.8% in round 3. Table 7 summarizes the main reasons for non-participation in each round of MDA. In the first round, more than half (54.3%) of non-participants were absent without providing a specific reason. Absenteeism continued to be a key factor in rounds two and three, with over one-third absent during each round. Reluctance to take ivermectin increased over successive rounds, with 22.3%, 45.1%, and 50.3% of non-participants citing unwillingness. Other reasons included illness, pregnancy, relocation, or mild adverse effects experienced in earlier rounds. Therefore, surveying a subset of participants from the target population may not fully capture the perspectives of the entire population.

**Table 7:** Reasons for not participating in each round of MDA.

Reason	Non-participation among eligible individuals					
	MDA-1 (*n* = 672)		MDA-2 (*n* = 951)		MDA-3 (*n* = 915)	
	*n*	%	*n*	%	*n*	%
Absent	365	54.3	375	39.4	321	35.1
Not willing	150	22.3	428	45.1	460	50.3
COVID-19	18	2.7	13	1.4	–	–
Death ^a^	2	0.3	4	0.4	5	0.5
Illness	45	6.7	45	4.7	42	4.6
Lactating	3	0.4	5	0.5	2	0.2
Moved out	63	9.4	32	3.4	31	3.4
Pregnancy	16	2.4	18	1.9	19	2.1
Repeated name	2	0.3	3	0.3	–	–
Unreachable	6	0.9	7	0.7	8	0.9
Side effect(s)	–	–	20	2.1	26	2.8
Withdrawal	–	–	1	0.1	1	0.1
No reason	2	0.3	–	–	–	–

^a^ not related to ivermectin MDA.

After excluding non-participants, over half (59.0%, *n* = 1,852) of eligible individuals completed all rounds of ivermectin MDA. An additional 15.0% (*n* = 471) completed two rounds, and 12.0% (*n* = 375) participated in just one round. In contrast, a small proportion (14.0%, *n* = 439) missed the entire campaign. Notably, while 5.1% and 8.9% completed the first round but missed the second or third rounds. Interestingly, a few (1.2–4.4%) participated in later rounds despite missing the first round of MDA (Table 8).

**Table 8:** Participation statuses among eligible samples in all rounds of MDA (*n* = 3,137).

MDA			*n*	%	Participation scenario
1st	2nd	3rd			
YES	YES	YES	1,852	59.0	Completed all three rounds of MDA
YES	YES	NO	159	5.1	Missed the third round only
YES	NO	YES	174	5.5	Missed the second round only
YES	NO	NO	280	8.9	Only participated in the first round
NO	NO	YES	58	1.8	Only participated in the third round
NO	YES	NO	37	1.2	Only participated in the second round
NO	YES	YES	138	4.4	Missed the first round only
NO	NO	NO	439	14.0	Did not participate in any round

Completed all three rounds of MDA [*n* = 1,852; 59.0%].

Completed exactly two rounds of MDA [*n* = 471 (159 + 174 + 138); 15.0%].

Completed only one round of MDA [*n* = 375 (280 + 58 + 37); 12.0%].

Did not participate in any round of MDA [*n* = 439; 14.0%].

## Discussion

The levels of KAP regarding diseases are key indicators that influence individuals’ adoption of preventive measures, early diagnosis, treatment compliance, and participation in public health interventions^30^. In this study, participants displayed above-average KAP levels, particularly in knowledge and attitudes toward malaria. These results are consistent with a previous study conducted in Thailand, which reported poor KAP levels among migrant and mobile populations^31^. These findings underscore the need for enhanced health promotion activities in the study locations to strengthen preventive measures and reduce malaria transmission. Some participants also held incorrect beliefs about malaria, such as the misconception that antimalarial medicines should not be taken with durian, a popular fruit in Thailand. This belief, which lacks scientific evidence, has been echoed in other studies, where a significant proportion of respondents avoided consuming fruit with medications^24^. Health promotion efforts should address such misconceptions, using actual survey findings to inform educational messages.

The present study also examines how general characteristics, such as age and occupation, influenced KAP regarding malaria. Participants aged 36–50 years demonstrated lower knowledge and practice scores, which could be attributed to their focus on earning family income through work that often distances them from routine health promotion activities^32,33^. Furthermore, the majority of participants were employed in forest-related occupations, making it difficult to consistently adhere to malaria preventive measures. These individuals face challenges such as working night shifts or in environments unsuitable for mosquito net use^34–36^. On the other hand, those working in non-forest-related settings often believed that malaria was only a risk in forested areas, potentially reflecting a public health focus on forest malaria that may have unintentionally led to the misconception that forests are the sole source of malaria risk, resulting in unfavorable attitudes toward malaria prevention^37^.

A study in Thailand found that only half of surveyed households possessed sufficient ITNs^10^and households with larger families often struggled to provide adequate bed nets. Individuals with a prior history of malaria or those who received health messages from healthcare providers or malaria volunteers demonstrated better KAP levels^38^. However, those without prior knowledge or exposure to malaria-related information displayed lower KAP levels. Therefore, community-based health promotion activities should be organized within villages or work sites, ensuring that both individuals with and without a history of malaria are included.

Nearly all participants in this study expressed a positive attitude toward the planned ivermectin MDA campaign. Those working in forest-related settings and those who believed in the possibility of malaria elimination in their villages demonstrated significantly higher acceptability rates. Participants recognized that malaria transmission is higher in the forested areas and were thus more inclined to follow healthcare providers’ advice and participate in MDA activities. These findings align with the Health Belief Model (HBM), which posits that individuals’ health-related behaviors are shaped by their perceptions of disease severity, the perceived benefits of the intervention, and their self-efficacy^29,39^. Accordingly, participants who believed malaria could be eliminated from their villages were more likely to accept the MDA campaign. While this study did not originally apply the HBM in its design, several key constructs such as perceived susceptibility to malaria, perceived severity, and perceived benefits of ivermectin MDA are conceptually aligned with participant responses. These constructs help interpret the reported acceptance levels and attitudes toward malaria prevention. Future research may benefit from incorporating these constructs more systematically to strengthen the behavioral science foundation of intervention design.

Despite ongoing efforts to control malaria through vector control measures in Thailand, such as ITNs and IRS, outdoor transmission remains a major challenge^40^. Low ITN usage^10^ increasing insecticide resistance^12,13^ and the heterogeneity of vector behavior have undermined the effectiveness of these interventions^20^. In response, the introduction of ivermectin MDA in southern Thailand represents an innovative approach. While reported acceptance was high, the actual completion rate of the MDA was lower among eligible participants. This large gaps can be attributed to several factors. First, social desirability bias may have influenced participants to report willingness to participate, particularly when surveyed by health-affiliated personnel. Second, real-world challenges such as labor migration, seasonal work, and temporary relocation, especially among forest-related workers, contributed to absenteeism. Third, mild side effects, fear of adverse reactions, or misinformation about ivermectin may have increased reluctance to participate in later rounds^24^. These findings highlight the importance of distinguishing between stated intentions and observed behaviors when planning and evaluating public health campaigns. Operational and logistical factors also influenced participant retention and compliance. The MDA rounds were spaced approximately one month apart, which may not have aligned with agricultural cycles and mobility patterns in the community. Some participants were unavailable due to seasonal labor demands, and there was no robust follow-up system in place to track defaulters or encourage continued participation. Moreover, the absence of personalized reminders or support mechanisms between rounds likely weakened adherence. Although ivermectin distribution was supported by healthcare staff and local malaria volunteers, systems for addressing absenteeism, tracking non-participants, or managing minor side effects were limited. Future MDA strategies may benefit from incorporating flexible scheduling, mobile health reminders, and targeted community engagement, especially for high-risk and mobile populations.

This study has several strengths and limitations. To our knowledge, it is the first to explore community acceptability of ivermectin MDA in Thailand. The findings provide valuable insights into community-specific factors that could inform the design of tailored interventions to improve participation. However, since ivermectin is a relatively new intervention to malaria control, the questionnaire did not include detailed information on its side effects, benefits, dosages, or treatment compliance. Additionally, the quantitative nature of the study limits a comprehensive understanding of the factors driving the high acceptability rates. Future qualitative research is needed to explore the underlying reasons behind community acceptability of ivermectin MDA and the challenges associated with its implementation, particularly in remote and high-transmission areas. The study focused on adult household heads or long-term residents, potentially limiting generalizability to younger adults, temporary migrants, or newly settled individuals who may have different risk profiles and health behaviors. Participation data from the entire eligible population were drawn from programmatic records maintained by village volunteers. Although these data were not part of the main survey sample, they provided valuable insights into real-world implementation. However, because they were not collected through structured interviews, they may not capture all underlying reasons for non-participation. The absence of qualitative data limits the ability to contextualize quantitative findings and explore participants’ nuanced perspectives. A mixed-methods approach would have enabled triangulation and richer interpretation of results. Despite these limitations, the study’s findings offer essential insights into improving ivermectin MDA participation rates and provide helpful guidance for malaria control programs in Thailand and other similar settings.

## Conclusions

This study highlights the need for improvements in malaria knowledge and attitudes, particularly among participants who scored poorly. Health promotion interventions should be tailored to individuals aged 36 to 50 and those with no or uncertain history of malaria. While participants demonstrated high relative acceptance of ivermectin MDA, the actual completion rate was lower than anticipated. The main reasons for non-participation were absence from the study area and reluctance to take ivermectin. To address these issues, community engagement initiatives are essential for encouraging eligible individuals, particularly those unwilling or absent without valid reasons, to complete the MDA. These efforts should focus on clearly communicating the risks and benefits of ivermectin, ensuring safety protocols are understood, and raising awareness about malaria prevention and control. Additionally, the study emphasizes the importance of intensively recruiting forest-related workers, who are at a higher risk of malaria exposure, to ensure high participation rates in the ivermectin MDA program.

## Data Availability

All data generated or analyzed for this study are included within the article. The de-identified raw dataset is available from the corresponding author upon reasonable request.

## Abbreviations

aOR: Adjusted odds ratio
CI: Confidence interval
GMS: Greater Mekong Subregion
HBM: Health belief model
IRS: Indoor residual spraying
ITN: Insecticide treated net
MDAL: Mass drug administration
WHO: World Health Organization
